# Top 100 cited articles on hemodialysis: A bibliometric analysis

**DOI:** 10.1097/MD.0000000000027237

**Published:** 2021-09-24

**Authors:** Yoo Jin Lee, Chang Min Heo, Sihyung Park, Yang Wook Kim, Jin Han Park, Il Hwan Kim, Junghae Ko, Bong Soo Park

**Affiliations:** Department of Internal Medicine, Inje University Haeundae Paik Hospital, Busan, Korea.

**Keywords:** analysis, hemodialysis, publications

## Abstract

**Introduction:**

This study was conducted to better understand hemodialysis by reviewing the most-cited articles related to it.

**Methods:**

We searched articles on the Web of Science and selected the 100 most frequently cited articles. Subsequently, we reviewed these articles and identified their characteristics.

**Results:**

The 100 most frequently cited articles were published in 21 journals. The majority of these papers were published in the following journals: *Kidney International* (26 articles), *New England Journal of Medicine* (18 articles), *Journal of the American Society of Nephrology* (14 articles), and the *American Journal of Kidney Disease* (13 articles). The 100 most-cited articles were published in 25 countries. The United States of America was the country with the highest number of publications (65 articles). The University of Michigan was the institution with the highest number of articles (14 articles). FK Port was the author with the largest number of publications (13 articles).

**Conclusions:**

This is the first study in the field of nephrology that provides a list of the 100 most-cited articles on hemodialysis. Through this study, clinicians will be able to recognize major academic interests and research trends in hemodialysis.

## 1 Introduction

A progressive stage of chronic kidney disease with a glomerular filtration rate of less than 15 mL/min/1.73 m^2^ is defined as end-stage renal disease (ESRD). ESRD is one of major global health problems. Its prevalence is gradually increasing worldwide and in Korea.^[1,2]^ ESRD patients often experience various complications including multiorgan dysfunction. In ESRD, hemodialysis (HD) and peritoneal dialysis are mainly used for renal replacement therapy. As both therapies have their advantages and disadvantages, the choice of the treatment method can be decided by the physical condition, patient's preference, or comorbidities. The number of patients requiring HD continues to increase annually. According to research by the National Health Insurance Service on medical aid, the number of HD patients increased by 31.9% from 44,136 in 2006 to 58,232 patients in 2010.^[3,4]^

The Science Citation Index was explained by 1964 at the Institute for Scientific Information. This was used to confirm that the academic contribution of the journal was high. It was also used to collect citation information and build indexes for database screening of scientific articles. Science Citation Index has become one of the most widely and frequently used databases for searching journals and assessing research outcomes. In the area of science, by increasing the number of articles, the availability of these articles in the form of compact discs or books was limited. This has resulted in a larger web version, known as the Web of Science: Science Citation Index Expanded.

The number of citations received by an article reflects the level of interest of the academic community in that particular topic. Therefore, a large number of citations indicate the significant impact of this article in the scientific community. The most frequently cited articles provide interesting insights into the process by which articles, subjects, and authors influence the field of research over time. By reviewing the most-cited articles, it can provide information on key areas of interest and avenues of research that have shown substantial growth in a specific field. Several researches have already analyzed the most frequently cited papers in several fields such as emergency medicine, general surgery, orthopedic surgery, plastic surgery, anesthesiology, obstetrics and gynecology, dermatology, critical-care medicine, and headache disorders.^[5–13]^ However, no research has previously analyzed the 100 most-cited articles on HD.

This study presents the most cited articles related to HD and aims to broaden the understanding of HD through it.

## 2 Methods

We conducted an analysis of the citation-related HD. The study was performed as follows: First, we only searched for HD-related articles, except for peritoneal dialysis-related articles. We searched the Web of Science (https://apps.webofknowledge.com) by restricting the document type to reviews and journal articles. The publication period was from 1969 to 2019. Articles meeting these criteria were sorted by the number of citations they had received.

Second, according to citation frequency, we selected 100 articles on HD. We then reviewed the contents of each article and classified them by the number of citations, year of publication, publishing journal, published country, authorship, and topic categories. The topic categories were organized as pathophysiology, epidemiology, survival and mortality, mineral metabolism, and vascular calcification. The first author was used as the criterion when there was more than 1 author. Recommendations were excluded from the study. No statistical techniques were used in this study. The data are presented as frequencies only. Since this study is an analysis of data from online databases and the privacy of patients will not be disclosed, so patients‘ informed consent and ethical approval are all not required.

## 3 Results

A total of 8941 HD-related articles were identified and analyzed. We chose the 100 most frequently cited papers and arranged them in descending order by the number of citations (Table 1). The most frequently cited paper was cited 1802 times, and the paper with the least number of citations was cited 322 times. Most of the papers (80 articles) received more than 360 citations.

**Table 1:** The top 100 cited articles about Hemodialysis.

Rank	Journal	Title	Number of citations
1	Journal of the American Society of Nephrology	Mineral metabolism, mortality, and morbidity in maintenance hemodialysis	1802
2	American Journal of Kidney Disease	Association of serum phosphorus and calcium × phosphate product with mortality risk in chronic hemodialysis patients: A national study	1778
3	New England Journal of Medicine	Atorvastatin in patients with type 2 diabetes mellitus undergoing hemodialysis	1710
4	New England Journal of Medicine	Accelerated atherosclerosis in prolonged maintenance hemodialysis	1642
5	Circulation	Impact of aortic stiffness on survival in end-stage renal disease	1632
6	American Journal of Kidney Disease	Death risk in hemodialysis-patients—the predictive value of commonly measured variables and an evaluation of death rate differences between facilities	1572
7	New England Journal of Medicine	The effects of normal as compared with low hematocrit values in patients with cardiac disease who are receiving hemodialysis and epoetin	1537
8	New England Journal of Medicine	Chronic hemodialysis using venipuncture and a surgically created arteriovenous fistula	1322
9	New England Journal of Medicine	Effect of dialysis dose and membrane flux in maintenance hemodialysis.	1234
10	New England Journal of Medicine	Rosuvastatin and cardiovascular events in patients undergoing hemodialysis	1214
11	Nephrology Dialysis Transplantation	Arterial media calcification in end-stage renal disease: impact on all-cause and cardiovascular mortality	1214
12	New England Journal of Medicine	Fibroblast growth factor 23 and mortality among patients undergoing hemodialysis	1140
13	Kidney International	Sevelamer attenuates the progression of coronary and aortic calcification in hemodialysis patients	1106
14	New England Journal of Medicine	The urea reduction ratio and serum albumin concentration as predictors of mortality in patients undergoing hemodialysis	1091
15	Lancet	Effect of human erythropoietin derived from recombination-DNA on the anemia of patients maintained by chronic-hemodialysis	1090
16	Journal of Clinical Investigation	Hemodialysis leukopenia—pulmonary vascular leukostasis resulting from complement activation by dialyzer	1034
17	Hypertension	Arterial calcifications, arterial stiffness, and cardiovascular risk in end-stage renal disease	1029
18	New England Journal of Medicine	Complement and leukocyte-medicated pulmonary dysfunction in hemodialysis	1014
19	Journal of The American Society of Nephrology	Association of elevated serum PO4, Ca × PO4 product, and parathyroid hormone with cardiac mortality risk in chronic hemodialysis patients	929
20	Biochemical and Biophysical Research Communications	A new form of amyloid protein associated with chronic-hemodialysis was identified as beta-2-microglobulin	850
21	Journal of The American College of Cardiology	Cardiac calcification in adult hemodialysis patients—a link between end-stage renal disease and cardiovascular disease?	835
22	New England Journal of Medicine	Sympathetic overactivity in patients with chronic renal failure	820
23	Nephrology Dialysis Transplantation	Arterial stiffening and vascular calcifications in end-stage renal disease	792
24	Lancet	Secondary prevention with antioxidants of cardiovascular disease in endstage renal disease (SPACE): randomised placebo-controlled trial	784
25	Circulation	Impact of aortic stiffness attenuation on survival of patients in end-stage renal failure	763
26	New England Journal of Medicine	Cinacalcet for secondary hyperparathyroidism in patients receiving hemodialysis	754
27	New England Journal of Medicine	Survival of patients undergoing hemodialysis with paricalcitol or calcitriol therapy	748
28	Kidney International	Survival predictability of time-varying indicators of bone disease in maintenance hemodialysis patients	671
29	Journal of The American Society of Nephrology	Adiponectin, metabolic risk factors, and cardiovascular events among patients with end-stage renal disease	652
30	Journal of The American Society of Nephrology	Activated injectable vitamin D and hemodialysis survival: a historical cohort study	647
31	American Journal of Kidney Disease	Electron beam computed tomography in the evaluation of cardiac calcifications in chronic dialysis patients	647
32	New England Journal of Medicine	In-center hemodialysis 6 times per week versus 3 times per week	630
33	Kidney International	Survival as an index of adequacy of dialysis	626
34	American Journal of Kidney Disease	Mortality risk for dialysis patients with different levels of serum calcium, phosphorus, and PTH: The dialysis outcomes and practice patterns	610
35	Transactions American Society For Artificial International Organs	Cannulation of blood vessels for prolonged hemodialysis	609
36	Kidney International	Vascular access use in Europe and the United States: results from the DOPPS	606
37	Kidney International	Effects of sevelamer and calcium on coronary artery calcification in patients new to hemodialysis	599
38	Hypertension	Central pulse pressure and mortality in end-stage renal disease	594
39	Kidney International	Vitamin D levels and early mortality among incident hemodialysis patients	592
40	Kidney International	Atherosclerotic cardiovascular disease risks in chronic hemodialysis patients	585
41	Kidney International	Predictors and consequences of altered mineral metabolism: the Dialysis Outcomes and Practice Patterns Study	556
42	Kidney International	Health-related quality of life as a predictor of mortality and hospitalization: the Dialysis Outcomes and Practice Patterns Study (DOPPS)	522
43	Journal of The American Society of Nephrology	Association of comorbid conditions and mortality in hemodialysis patients in Europe, Japan, and the United States: the dialysis outcomes and practice patterns study (DOPPS)	511
44	Kidney International	Type of vascular access and mortality in US hemodialysis patients	508
45	New England Journal of Medicine	Daily hemodialysis and the outcome of acute renal failure	507
46	American Journal of Kidney Disease	A malnutrition-inflammation score is correlated with morbidity and mortality in maintenance hemodialysis patients	497
47	Kidney International	U curve association of blood pressure and mortality in hemodialysis patients	488
48	Nephron	Mortality risk-factors in patients treated by chronic-hemodialysis—report of the diaphand collaborative study	488
49	Kidney International	Increasing arteriovenous fistulas in hemodialysis patients: problems and solutions	484
50	American Journal of Kidney Disease	Reevaluation of risks associated with hyperphosphatemia and hyperparathyroidism in dialysis patients: Recommendations for a change in management	479
51	Hypertension	Carotid arterial stiffness as a predictor of cardiovascular and all-cause mortality in end-stage renal disease	479
52	American Journal of Kidney Disease	Canadian hemodialysis morbidity study	473
53	JAMA- Journal of the American Medical Association	Effect of clopidogrel on early failure of arteriovenous fistulas for hemodialysis—a randomized controlled trial	465
54	Journal of The American Society of Nephrology	Hematocrit level and associated mortality in hemodialysis patients	458
55	Kidney International	The dose of hemodialysis and patient mortality	450
56	New England Journal of Medicine	Infection with hepatitis GB virus C in patients on maintenance hemodialysis	446
57	Annals of Surgery	Vascular access for hemodialysis—patency rates and results of revision	443
58	American Journal of Kidney Disease	Interleukin-6 predicts hypoalbuminemia, hypocholesterolemia, and mortality in hemodialysis patients	437
59	BMJ-British Medical Journal	Association between recombinant human erythropoietin and quality of life and exercise capacity of patients receiving haemodialysis	434
60	New England Journal of Medicine	Hepatitis-B vaccine in patients receiving hemodialysis— immunogenicity and efficacy	426
61	Kidney International	Cardiac and arterial interactions in end-stage renal disease	420
62	Journal of The American Society of Nephrology	Arterial calcifications and bone histomorphometry in end-stage renal disease	408
63	Circulation	Predictive value of cardiac troponin I and T for subsequent death in end-stage renal disease	403
64	Journal of The American Society of Nephrology	EPIBACDIAL: a multicenter prospective study of risk factors for bacteremia in chronic hemodialysis patients	399
65	Kidney International	Aortic pulse wave velocity index and mortality in end-stage renal disease	392
66	Circulation	Plasma norepinephrine predicts survival and incident cardiovascular events in patients with end-stage renal disease	391
67	Kidney International	Immunologic function and survival in hemodialysis patients	388
68	American Journal of Kidney Disease	Hemodialysis patient-assessed functional health status predicts continued survival, hospitalization, and dialysis-attendance compliance	387
69	Kidney International	Multiple measurements of depression predict mortality in a longitudinal study of chronic hemodialysis outpatients	383
70	Annals of Internal Medicine	Catheter-related bacteremia and outcome of attempted catheter salvage in patients undergoing hemodialysis	382
71	Journal of Clinical Investigation	Beta(2)-microglobulin modified with advanced glycation end-products is a major component of hemodialysis-associated amyloidosis	381
72	Journal of Vascular Surgery	A strategy for increasing use of autogenous hemodialysis access procedures: impact of preoperative noninvasive evaluation	380
73	Clinical Journal of The American Society Of Nephrology	Hemodialysis-induced cardiac injury: determinants and associated outcomes	374
74	Kidney International	Effects of sevelamer and calcium-based phosphate binders on mortality in hemodialysis patients	371
75	Transactions American Society For Artificial International Organs	Syndrome of dyspraxia and multifocal seizures associated with chronic hemodialysis	371
76	New England Journal of Medicine	Staphylococcus-areus nasal carriage and infection in patients on hemodialysis—efficacy of antibiotic	370
77	Nephrology Dialysis Transplantation	Vascular access use and outcomes: an international perspective from the dialysis outcomes and practice patterns study	369
78	Kidney International	Hemodialysis-associated hypotension as an independent risk factor for 2-yr mortality in hemodialysis patients	369
79	JAMA- Journal of the American Medical Association	The quality of life of hemodialysis recipients treated with recombinant human erythropoietin	369
80	Kidney International	Depression as a predictor of mortality and hospitalization among hemodialysis patients in the United States and Europe	366
81	Neurology	Cognitive impairment in hemodialysis patients is common	362
82	Journal of The American Society of Nephrology	Diabetes mellitus, aortic stiffness, and cardiovascular mortality in end-stage renal disease	359
83	American Journal of Kidney Disease	Anemia management and outcomes from 12 countries in the Dialysis Outcomes and Practice Patterns Study (DOPPS)	357
84	Kidney International	Influence of uremia and hemodialysis on circulating interleukin-1 and tumor necrosis factor-alpha	357
85	Kidney International	Prevention of hemodialysis fistula thrombosis—early detection of venous stenosis	353
86	American Journal of Kidney Disease	Simple nutritional indicators as independent predictors of mortality in hemodialysis patients	349
87	American Journal of Kidney Disease	Predialysis blood pressure and mortality risk in a national sample of maintenance hemodialysis patients	346
88	Journal of Clinical Pathology	Acquired cystic-disease of kidneys – hazard of long-term intermittent maintenance hemodialysis	344
89	Journal of The American Society of Nephrology	Alterations of left ventricular hypertrophy in and survival of patients receiving hemodialysis: follow-up of an interventional study	343
90	Kidney International	Vascular access and increased risk of death among hemodialysis patients	340
91	Kidney International	Influence of excess weight on mortality and hospital stay in 1346 hemodialysis patients	339
92	New England Journal of Medicine	Use of a *Staphylococcus aureus* conjugate vaccine in patients receiving hemodialysis.	338
93	Kidney International	Cardiac diseases in maintenance hemodialysis patients: results of the HEMO Study	332
94	Kidney International	Anaphylatoxin formation during hemodialysis—effects of different dialyzer membranes	331
95	Journal of The American Society of Nephrology	Effects of body size and body composition on survival in hemodialysis patients	330
96	Journal of Clinical Investigation	Involvement of beta(2)-microglobulin modified with advanced glycation end-products in the pathogenesis of hemodialysis-associated amyloidosis—induction of human monocyte chemotaxis and macrophage secretion of tumor-necrosis-factor-alpha and interleukin-1	329
97	American Journal of Kidney Disease	Reduction in recombinant-human erythropoietin doses by the use of chronic intravenous iron supplementation	327
98	Journal of The American Society of Nephrology	High-efficiency postdilution Online hemodiafiltration reduces all-cause mortality in hemodialysis patients	325
99	Journal of The American Society of Nephrology	Mineral metabolism and arterial functions in end-stage renal disease: potential role of 25-hydroxyvitamin D deficiency	324
100	Journal of The American Society of Nephrology	Association among SF36 quality of life measures and nutrition, hospitalization, and mortality in hemodialysis	322

DNA = deoxyribonucleic acid, DOPPS = the Dialysis Outcomes and Practice Patterns Study, HEMO = hemodialysis, PTH = parathyroid hormone.

The 100 most-cited articles were published in 21 journals. Of the 21 journals, the one with the highest number of the most cited articles was *Kidney International* (26 articles), followed by *New England Journal of Medicine* (18 articles), *Journal of the American Society of Nephrology* (14 articles), and *the American Journal of Kidney Disease* (13 articles). Over half of these articles were published in these 4 journals (58 articles) (Table 2).

**Table 2:** Journals with 3 or more of the top 100 cited articles on hemodialysis.

Rank	Journal	Number of articles
1	Kidney International	26
2	New England Journal Of Medicine	18
3	Journal Of The American Society of Nephrology	14
4	American Journal Of Kidney Disease	13
5	Circulation	4
6	Hypertension	3
7	Journal Of Clinical Investigation	3
8	Nephrology Dialysis Transplantation	3

These studies were published in 25 countries. The United States of America had the highest number of publications (65 articles), followed by France (20 articles), Japan (14 articles), Germany (10 articles), and Canada (8 articles) (Table 3).

**Table 3:** Countries with 3 or more of the top 100 cited articles on hemodialysis.

Rank	Country	Number of articles
1	United States of America	62
2	France	16
3	Japan	6
4	Canada	4
5	Germany	3

**Figure 1:**
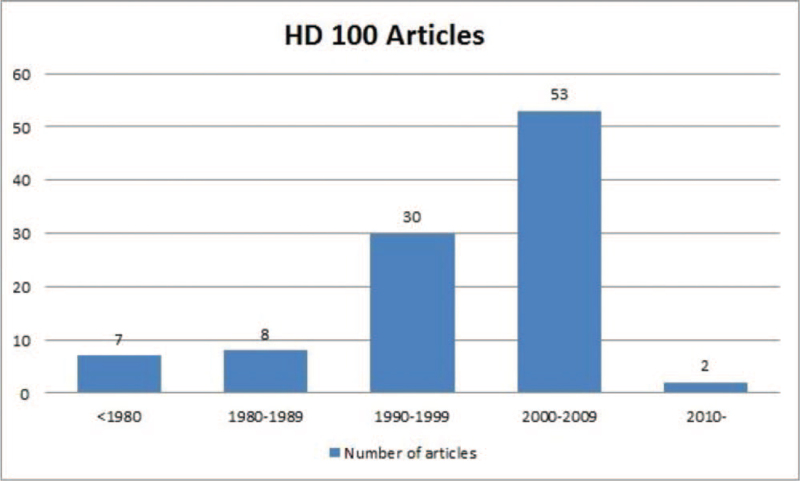
Number of the 100 most cited articles on hemodialysis according to decade.

The decades during which these articles were published are shown in Figure 1. Of all the articles, 55 were published in the past 2 decades. The earliest published article was published in 1966, while the most recently published article was published in 2013. More than 60 of the 100 most cited articles were provided by 17 institutions (Table 4). In articles with multiple authors, counting was based on the institution of the first author. The University of Michigan (14 articles) and the University of Michigan System (14 articles) were the institutions that published the highest number of articles, followed by the Assistance Publique Hopitaux Paris (12 articles) and the University of California system (12 articles).

**Table 4:** Institutions of the first author's origin which have 4 or more of the top 100 cited articles on hemodialysis.

Rank	Institution	Number of articles
1	University of Michigan	9
2	Hôpital F.H. Manhès, Fleury-Mérogis	7
3	University of California San Francisco	5
4	Harvard Medical School	5
5	Denver Nephrology Associates	5
6	University of Minnesota	4

The top authors who have published more than 4 articles on HD are listed in Table 5. FK Port was the author who published the most number of articles (13 articles).

**Table 5:** First authors with 4 or more of the top 100 cited articles on hemodialysis.

Rank	First author	Number of articles
1	Block, GA	5
2	London, GM	5
3	Blacher, J	4

## 4 Discussion

In this article, we searched and reviewed the 100 most-cited articles on HD. These articles provided advanced insights on scientific perspectives and progress in the field of HD.

The most-cited article was published by the *Journal of the American Society of Nephrology* in 2004 and was written by the Block.^[14]^ To identify associations between mineral metabolism disorders (hypercalcemia, hyperphosphatemia, and secondary hyperparathyroidism), mortality, and morbidity in HD patients, a nationally representative database of >40,000 HD patients was analyzed. This study showed strong associations between higher concentrations of serum calcium and phosphorus and higher mortality. They also found associations between hyperphosphatemia and hyperparathyroidism and cardiovascular, fracture, and all-cause hospitalization. These results support the hypothesis that mineral metabolism disorders are associated with the risk of cardiovascular disease in ESRD patients.

The second most cited article was published by the *American Journal of Kidney Disease*, entitled “Association of serum phosphorus and Ca × PO_4_ product with mortality risk in chronic HD patients: A national study.”^[15]^ The goal of this study was to estimate the level to which serum phosphorus is maintained in 2 large national, random samples of patients who have been receiving HD for at least 1 year. Ca × PO_4_ product levels above 72 mg^2^/dL^2^ were observed in 20% of the patients and were associated with a higher relative risk of death compared with those with a Ca × PO_4_ product between 42 and 52 mg^2^/dL^2^. These results support that intensive control of hyperphosphatemia can increase the survival rate of patients.

The most frequent topic discussed in these articles was cardiovascular mortality in hemodialysis patients (22 articles). The most cited article about cardiovascular mortality in HD patients was authored by Lindner in 1974 and published by the *New England Journal of Medicine*.^[16]^ This study reviewed mortality and morbidity due to cardiovascular complications in long-term HD patients in Seattle. The results showed that the incidence of arteriosclerotic complications was several times higher in this group than in the normal and hypertensive groups of comparable age, and was similar to the rate of cardiovascular complications found in patients with type 2 hyperlipoproteinemia. These outcomes indicate that increased atherosclerosis is a major risk for patients on long-term maintenance HD. The second most frequently discussed topic was chronic kidney disease-mineral and bone disorder in HD patients (15 articles). The articles about this topic provided information about laboratory changes in mineral metabolism during HD and mortality risks in chronic HD patients.

We also found some interesting trends among the subjects of the articles owing to the fact that they kept on changing from decade to decade. First, pathophysiology was the most frequently covered subject. The main key words related to this topic are: uremia, electrolyte, mineral metabolism. This was similar to the results of analyses performed in other fields.^[4,5,7,12]^ Other subjects often covered in articles were treatment and basic research. Second, an increasing number of articles were published as time passed from the 1990s to the 2010s. From 2000 to 2009, 53 of the 100 most-cited articles were published. Papers related to Chronic Kidney Disease-Mineral and Bone Disorder were more common before the 2000s, whereas papers on more diverse topics were published after the 2000s. Articles on the most common topic, cardiovascular mortality in HD patients, were published mainly after the 2000s. The main key words related to this topic are: atherosclerosis, vascular calcification. This trend is thought to be because of the higher concern regarding long-term complications, mortality, and morbidity in patients receiving maintenance HD.

Sixty-five articles were published in the United States, while 20 articles were published in France. In other areas where similar studies were conducted, the highest number of papers was published in the United States.^[4–7,9–12]^*Kidney International* published the highest number of the most cited articles (26 articles), followed by the *New England Journal of Medicine* (18 articles). American institutions have made significant contributions to the advancement of HD research. This is because the American scientific community can conduct research with enormous financial resources. Moreover, American writers prefer to publish their research in easily accessible American journals and usually cite papers written in English.^[17]^

We found that none of the 100 most cited papers originated in Africa. This may be due to the difficulties in accessing information, conducting and publishing research, and the language barrier experienced by researchers in Africa.

Our study has some inherent limitations. This research was conducted because of the controversy regarding the value of citations. The number of citations does not reflect whether the study was referenced in a positive or negative way.^[18]^ The papers cited most frequently may not necessarily be the most important and meaningful one.^[19]^ Certain types of articles, such as meta-analyses, systematic reviews, and guidelines tend to be cited more than others.^[20]^ In addition, older papers tend to be cited more frequently. However, evaluating the number of citations is a better way to assess the advantages of a paper. Analysis about citation rate is able to prove the advancement in a particular field of expert knowledge and give a retrospective aspect of scientific development.^[21]^

## 5 Conclusions

This is the first study in the field of nephrology to provide a list of the 100 most-cited articles on HD. This study provides major academic interest and research trends related to HD.

## Author contributions

**Conceptualization:** Sihyung Park, Bong Soo Park.

**Data curation:** Yoo Jin Lee, Sihyung Park.

**Formal analysis:** Junghae Ko.

**Investigation:** Jin Han Park, Il Hwan Kim, Junghae Ko.

**Project administration:** Yang Wook Kim.

**Supervision:** Yang Wook Kim, Bong Soo Park.

**Validation:** Jin Han Park, Il Hwan Kim.

**Visualization:** Jin Han Park, Il Hwan Kim.

**Writing – original draft:** Chang Min Heo.

**Writing – review & editing:** Chang Min Heo, Yoo Jin Lee, Bong Soo Park.
